# Critical Factors in the Implementation of Risk Awareness Education in Universities in China

**DOI:** 10.3389/fpsyg.2022.803360

**Published:** 2022-08-23

**Authors:** Ling Liu, Xiaoge Pei, Yingchun Han, Xiaoling Liao

**Affiliations:** ^1^School of Marxism, School of Public Policy and Administration, Nanchang University, Nanchang, China; ^2^Faculty of Social Sciences and Liberal Arts, UCSI University, Kuala Lumpur, Malaysia; ^3^Mental Health Center, Luoyang Normal University, Luoyang, China; ^4^School of Marxism, Nanchang University, Nanchang, China; ^5^School of Economics and Management, Nanchang University, Nanchang, China

**Keywords:** universities in China, campus risk, risk awareness education, critical factor, curriculum and instruction

## Abstract

Under the influence of social changes, latent factors in campus safety are increasing, and dealing with them is becoming more difficult. Facing the challenges in the pluralistic society, students need to cope with the changes of external and internal environments in the dynamic society. Additionally, there are new events on campus at any time, which may lead to campus risk. The frequent events that have occurred on campus in recent years have created difficulties for school administrative units. Implementing campus risk management strategies and conducting risk awareness education campaigns are, therefore, necessary. The fact that we are in a technologically dynamic age is another factor that makes risk awareness and proper risk management essential for individual survival and sustainable development of organizations. The participants of the study were university students in Nanchang, Jiangxi Province. Three hundred copies of the questionnaire were distributed, and 238 valid copies were retrieved, representing a retrieval rate of 79%. The results of the survey show that “life education” is the most emphasized dimension, followed by “curriculum and instruction,” and “environmental planning.” The five most emphasized indicators among the 14 indicators are opportunity education, physical activity, role-play, team competition, and learning area planning. The results suggest that school administrative units can take control in an emergency and reduce the likelihood of school members being threatened or harmed by the risk factor, and educators can make a quick decision to turn risk into opportunity.

## Introduction

A campus that is considered peaceful may hide a great potential for risk. As society changes, the latent factors for campus safety are increasing at the national level, and dealing with them is becoming more and more difficult. Facing the challenges in the pluralistic society, students must cope with the changes of external and internal environments in the dynamic society. Additionally, there are new events on campus at any time, which may lead to campus risk. Maslow placed physiological needs at the bottom of the hierarchy, and then, safety needs, which means that people had to satisfy the lowest physiological needs, including water, food, sleep, and homeostasis, before they could satisfy the safety needs, i.e., personal safety, for survival. The safety needs are urgently needed after the basic physiological needs were satisfied (Öznacar, 2018). The basic survival needs included physical safety and psychological safety, so a lack of safety in life would not satisfy other needs beyond safety needs and could have great threats and effects on personal survival. Safety was the fundamental survival need, but people applied different standards to “safety.” In a negative sense, safety meant having no hazards or accidents and being safe. Positively, safety meant seeking and applying viable strategies to avoid accidents and achieve safety (Bilgin and Öznacar, 2017). Consequently, the implementation of risk management strategies on campus and risk awareness education is necessary. The fact that we are in a technologically dynamic age is another factor that makes risk awareness and proper risk management essential for individual survival and the sustainable development of organizations.

As for the latent factors of campus safety, people, relationships, time, places, and objects on- and off-campus should be highlighted. For students, student safety was based on their concept of safety, knowledge, attitude, and behavior. In summary, student safety refers to safety management in the students’ environment, protection, and prevention of possible accidents, teaching relevant safety knowledge to students, and creating a safe, warm, and harmonious learning environment (Aspiranti et al., 2011). Based on the above opinions, one can understand the importance of safety for people. Positive prevention measures should be taken for students based on the idea that prevention is better than cure to reduce the probability of emergencies and accidents. The importance and measures of safety should be promoted in time from students’ life experience to learn the proper safety concept and behavior, cultivate the ability of safety analysis and prediction of hazards in the environment, and reduce unrealistic risk behaviors to ensure safety (Welch, 2018). The possibility of potential dangers in each of these components should not be ignored, and the continuous improvement of vigilance can help to face a danger calmly and not panic in case of serious injury. Schools used to be organizations with higher internal stability and fewer extrinsic factors; however, in this case, school educators often lacked the awareness of unexpected developments, believing the low likelihood of major events on campus. In addition, the campus is a very loosely organized place, and many risk events on campus are traceable before they break out. Frontline education personnel should, therefore, have a thorough understanding of the causes of campus risk events to take preventive measures in advance. This study discusses factors in the implementation of risk awareness training in Chinese universities, with the expectation that school administrative units can take control and reduce the likelihood of school members being threatened or harmed by the risk factor and that educators can make a quick decision to turn risk into opportunities, which are the critical roles.

## Literature Review and Hypothesis

### Campus Risk

Kim et al. (2018) considered safety as the basic need of an individual and the cornerstone of an organization, from individual health to the survival and development of the world. Nam (2017) considered that any event that causes a school to temporarily stop its activities can be a campus risk. Schools need to be alert and find immediate solutions to maintain the usual school operations. Chary et al. (2019) referred to campus risk as risk events that occur on campus. School faculty and staff, as well as students, are the assets under care when taking action. Campus risks are unsafe or unexpected situations, events occurring on- or off-campus that affect the normal operation of the school organization or seriously threaten the psychological or physiological health of teachers and students and require effective control and management within a short period of time. Hernandez-Suarez et al. (2019) defined “campus risk” as any event or situation that occurs on campus or is associated with campus members that result in disruption, pressure, and injury to school members and cannot be effectively and immediately resolved with existing school manpower and resources. Alpi and Evans (2019) regarded it as events that occur on- or off-campus that result in faculty, staff, and students being at risk due to the factors regarding school safety. Classroom accidents, natural disasters, faculty and staff misconduct, severe corporal punishment, and major conflicts even affect school operations. In terms of who is affected, López et al. (2018) noted that teachers and students in schools can experience the greatest impact, but communities and even the public can also be affected. Unlike other organizations, a school is a place of care and instruction that cannot be closed simply because of risk events.

### Risk Awareness Education

Hetu et al. (2018) pointed out that it is the responsibility of caregivers to provide students with safety-related knowledge. Next to family, school is the most important interaction and learning place for students. In this respect, parents and teachers are responsible for protecting students and providing them with knowledge about their safety. Paulik et al. (2019) suggested teaching students to risk awareness to develop a deeper understanding of accidental injuries, understand how to protect themselves when in danger, pay attention to safety measures in life, reduce safety problems caused by human negligence or mistakes, and develop the habit of taking precautions. Bellamy et al. (2019) explained the importance of risk awareness training as guiding students to effectively and correctly assess the whole environment, helping them to develop a deeper understanding of living with the habit of safety and promoting the understanding and prevention of accidental injuries according to safety cognition and alertness. Lin et al. (2017) mentioned that risk awareness education aims to prevent the occurrence of accidental injuries and to teach students to ensure physical and psychological safety, as well as to avoid unwanted or accidental injuries. Nickerson et al. (2019) stated that risk awareness education does neither mean threatening children nor does it expect children to learn how to get out of danger after being hurt, but it emphasizes and encourages a positive attitude toward establishing safety, uses positive communication, and protects them by setting an example. Gunawan et al. (2019) indicated that the importance and objective of risk awareness education are to prevent students from unexpected injuries and to maintain their physical and psychological safety, as well as to teach them to understand accidental injuries, pay attention to the environment, and avoid danger.

### Factors in Risk Awareness Education

Young et al. (2017) conducted a study on risk awareness education and divided the strategies commonly used in teachers’ practice in risk awareness education into three areas: environmental planning, curriculum and instruction, and life education. These applications are described below.

#### Environmental Planning

(1) Planning a safe classroom: This dimension refers to keeping the classroom environment healthy and clean and paying attention to ventilation systems, smooth movement, good lighting, comfortable temperature, and adjusting classroom decoration to each subject. (2) Planning a specific activity area: Teachers plan different activity areas and places, e.g., the location near the whiteboard, sticking lines on the floor, arranging students’ seats and activity area during classroom events, arranging table manners, planning nap space, and removing dangerous materials from the environment. (3) Planning a suitable learning area: This dimension refers to clearly defining the boundaries of each learning area in students’ learning environment, establishing an appropriate activity and allocating a space of the same size for each learning area, separating dynamic and quiet learning areas, developing learning area’s play rules and other related rules to be discussed between teachers and students, and understanding students’ use and boundaries of the learning area, as well. (4) Examining the safety of facilities and teaching aids: Facilities and teaching aids in the area must meet safety standards. Teachers and caregivers should regularly review the conditions of use of the facilities for safety and should make sure that the teaching aids are appropriate for students’ age. Planning for a safe environment can reduce the number of accidents, but flexibility for change allows students to learn in a good and safe environment (Pranata et al., 2019).

#### Curriculum and Instruction

(1) Group discussion: During classroom activities, teachers can provide students with risk awareness through group discussion, setting rules, autonomous class rules, and instructions to ensure that students understand safety-related knowledge, can develop sensitivity to environmental problems, can recognize possible risks in the living environment, and can establish positive behavior habits to reduce unsafe behaviors, as well as can let students gain insight into the different ideas through group discussion to improve the safety-related awareness and learn self-protection methods and resilience. (2) Exercise: Teaching students escape methods and proper behavior in the event of an earthquake or other disaster, teachers can show children the horror of disasters and show them the escape method and route to escape in school to deepen children’s perspective through role-plays and exercises. (3) Games and team competitions: Students enjoy games. The most important activity from the perspective of school children is “play” because games and imagination are the best teaching tools for them. (4) The use of music, song, and dance: Teachers can use music, song, and dance as media to support the instructional processes of risk education. For example, they can inform children about activities by starting with music or attract students’ attention by wrapping the content of risk education in interesting songs or accompanying it with dance movements to deliver the lesson and promote effective teaching. (5) The use of picture books and cards: Stories or situations are important ways for preschool children to acquire knowledge and experience because this teaching method can promote language development and increase imagination and creativity. (6) The use of audiovisual instructional media: Teachers can use various audiovisual equipment, such as films, educational videos, and radios, to teach students about the concept of safety and promote safety cognition, as well as to discuss possible responses to an accident together.

### Life Education

Instructional strategies in life education include the followings. (1) Teachers setting an example: Using examples is better than abstract instructions. Students in the learning phase would like to imitate the language or behavior of adults, and teachers are the role-model in schools. (2) Student role-play: Teachers can initiate risk awareness education through role-playing and use peer relations, e.g., providing models and having students assist in the instructional processes to give children a specific and permanent learning paradigm to learn proper safety behaviors and further cultivate safety habits. (3) Prior individual counseling and opportunity education: When children have unsafe behavior or reveal some behaviors related to safety, teachers can discuss with students about safety issues through individual guidance or opportunity education. They can teach them proper risk awareness and the right safety knowledge and behavior. (4) The use of picture prompt strategy: Teachers can use picture prompts in risk awareness education, such as turning arrangements created collaboratively by teachers and students into pictures or displaying pictures as posters to decorate the environment, so that students can see them at any time to promote peripheral learning. (5) Application of demonstration prompt strategy: During risk awareness education, students should know and understand the safety factors, and the learning objectives can be demonstrated and taught through opportunity education. (6) The use of reinforcement strategy: Teachers usually apply reinforcement strategies to support the teaching of risk awareness education, including concrete rewards or encouragement and praise (Takahashi et al., 2018).

## Methodology

### Research Method

Turner (2017) pointed out the common methods to confirm critical success factors as (1) regression analysis, (2) factor analysis, (3) Delphi method, and (4) Analytic Hierarchy Process (AHP). Brian (2017) suggested using the Analytic Hierarchy Process to collect the opinions of scholars, experts, and participants through group discussions to simplify complicated problems into simple elements and to calculate the contribution or priority of compositions in a hierarchy that corresponds to the elements in the previous hierarchy. Sindhu et al. (2017) explained after the objective interview with the relevant department supervisors that the goals and tasks are first confirmed based on the management program. They added that the individual critical success factors are then proposed according to the individual’s practical experience and needs. The critical success factors to achieve the goal are selected, organized, and sequenced based on analyses so that resources can be effectively distributed in the critical factors. Finally, indicators are established for the effectiveness of the practice.

An expert opinion survey was conducted in this study. Given the problems of mean, decision attributes, and group decisions, inaccuracies in the traditional Delphi method, the Fuzzy Delphi method (FDM), and the Analytic Hierarchy Process (AHP) were used for the data analysis in this study to determine the precise critical factors in the practice of risk awareness education of universities in China.

Analytic Hierarchy Process (AHP): After integrating expert opinions, a hierarchical system was constructed from complicated decision-making systems developed through hierarchies to clarify problems. Various dual judgments were complemented by pairwise comparisons to evaluate the importance of factor weights.

### Establishment of Indicator

The questionnaires in this study were emailed to experts in various fields. The initial expert feedback was organized by considering universities in China that teach risk awareness education. Such considerations with similar properties were classified and sent back to the experts to get their opinions. After several rounds of email inquiries, the major classification was achieved. An expert meeting was called to discuss the critical factors in universities in China, which include risk awareness education, environmental planning, curriculum and instruction, and life education. These critical factors were used as AHP dimensions, and the corresponding classifications were used as the basis for creating the AHP questionnaire. The following research principles were revised using the Delphi method.

(1) Environmental planning: classroom planning, activity area planning, learning area planning, facility examination, teaching, and safety.
(2) Curriculum and instruction: group discussions, exercises, team competitions, the use of teaching materials, and audio-visual media.
(3) Life education: role modeling, role-playing, opportunity education, and instructional strategies.

### Research Objective

In recent years, with the rapid development of society and economy in China, students are exposed to an increasingly harsh social environment and enormous pressure in terms of learning, life, adjustment, emotion, and employment. Students might easily face psychological problems and even experience serious psychological risks. In this case, universities should constantly teach risk awareness to strengthen students’ risk immunity.

With the participation of university students in Nanchang, Jiangxi Province, this study investigates public risk awareness, prevention and response ability, and public risk awareness education in universities. A total of 300 copies of the questionnaire were distributed, and 238 valid copies were retrieved, representing a retrieval rate of 79%.

## Results

After completing hierarchical weighing, the distribution was calculated based on the relative importance of the indicators in various hierarchies to reveal the importance of the indicators in the entire system and the overall weight of factors in the universities in China that conduct risk awareness education was calculated (Table 1).

**TABLE 1:** Overall weight of factors in risk awareness education in universities in China.

Dimension	Hierarchy 2 weight	Hierarchy 2 sequence	Indicator	Overall weight	Overall sequence
Environmental planning	0.258	3	Classroom planning	0.046	11
			Activity area planning	0.066	8
			Learning area planning	0.088	5
			Facility examination	0.030	14
			Teaching aid safety	0.034	13
Curriculum and instruction	0.335	2	Group discussion	0.040	12
			Exercise	0.108	2
			Team competition	0.095	4
			Application of teaching material	0.074	7
			Audiovisual media	0.050	10
Life education	0.407	1	Making oneself an example	0.055	9
			Role-play	0.100	3
			Opportunity education	0.131	1
			Instructional strategies	0.083	6

## Discussion

The administrative units in universities can apply life education during the orientation training of teachers and caregivers so that they can teach students about the possible risks and implement the curriculum to instruct students about the thematic order of risks for the training of risk awareness education’s practice strategies. After the training, the effectiveness of learning the contents of risk awareness education should be reviewed so that teachers can gain professional knowledge and skills related to risk management to implement on campus as needed. Disaster prevention exercises should be reinforced and implemented so that students can remember how to cope with risks and disasters and can respond calmly to sudden risks and disasters. Teachers can try different instructional strategies, utilize community resources, and invite experts, teachers, and caregivers for the cooperation to teach risk awareness using the appropriate methods, in which students are interested to deepen their impression, allow them to have multiple experiences, and implement their safety knowledge. Educational administrative units can compile booklets on crisis awareness education (Öznacar and Dagli, 2019). These can be illustrated with short and concise text and graphics so that they are easy for teachers to read and remember (Cowan and Rossen, 2013). This can enrich teachers’ pedagogical knowledge of the field and inform crisis awareness education (Mulvaney et al., 2012). In terms of designing the curriculum for students’ risk awareness education, administrative units in universities can enhance teaching and training for the application of group discussions, games, and picture books or cards. Furthermore, thematic training on risk awareness education can be held to help the teachers improve the practices of group discussion, games, and picture books or cards to meet the needs of students in the teaching environments and to strengthen the implementation of students’ risk awareness education. In addition, teachers can ask, discuss with, or learn from other teachers with different experience backgrounds to build collaboration. Sharing experiences, exchanging knowledge, and reflecting after practice can promote professional knowledge and skills in risk awareness education and facilitate implementation in the classroom. It is also important to ensure that teachers implement crisis awareness education. With this in mind, field research and academic practices in crisis awareness education can be used to examine how the teaching is occurring in the field. Teachers can understand the importance of crisis awareness education through appropriate supervision, evaluation, and assessment (Bosworth et al., 2011). Since schoolteachers are busy with their coursework and school administrative matters, the unfamiliarity with the content of crisis management training on campus would lead to the neglect of crisis management on campus (Özdemir, 2012; Estep, 2013). In this case, the higher education administrative unit can establish a special crisis office to implement a specific division of labor, review the adequacy of schools’ crisis awareness education programs, and ensure the smooth operation of crisis teams. Regular dissemination of crisis awareness education at schools and sharing experience would have more real effects than schools working behind closed doors. Besides the actual needs of schools to be reviewed and depending on the promotion of crisis awareness education in schools, the budget should be adjusted accordingly to support the promotion of crisis education on campus and various crisis education activities (Howat et al., 2012; Lalonde and Roux-Dufort, 2013).

## Conclusion

In this study, the AHP was used to analyze the data. The nature of the interaction between dimensions through the ultimate hierarchy was observed. Accordingly, the nature of the impact of each dimension on the alternatives was determined. Accordingly, the following conclusions were drawn from the results of the empirical analysis.

Among the dimensions in Hierarchy 2, “life education,” was the most emphasized dimension with a weight of 0.407, about 40.7% of overall weight, followed by “curriculum and instruction” (weighted 0.335), and “environmental planning” (weighted 0.258). Accordingly, risk awareness education was the most emphasized dimension for universities in China implementing life education.

For the indicators of the dimensions in Hierarchy 3, the hierarchical weights were sequenced as below (Table 2).

**TABLE 2:** Weights in Hierarchy 3.

Dimension	Indicator	Overall weight	Hierarchy sequence
Environmental planning	Classroom planning	0.046	3
	Activity area planning	0.066	2
	Learning area planning	0.088	1
	Facility examination	0.030	5
	Teaching aid safety	0.034	4
Curriculum and instruction	Group discussions	0.040	5
	Exercises	0.108	1
	Team competitions	0.095	2
	Application of teaching materials	0.074	3
	Audio-visual media	0.050	4
Life education	Role modeling	0.055	4
	Role-play	0.100	2
	Opportunity education	0.131	1
	Instructional strategies	0.083	3

1. Indicators in environmental planning were ordered as learning area planning, activity area planning, classroom planning, teaching aid safety, and facility examination.
2. Indicators in curriculum and instruction were sequenced as exercises, team competitions, application of teaching materials, audio-visual media, and group discussions.
3. Indicators in life education were sequenced as opportunity education, role-play, instructional strategies, and role modeling.

Based on the weighing of critical factors in universities in the implementation of risk awareness education in Chinese universities, the top five indicators (Table 3) were ranked as opportunity education, exercise, role-play, team competition, and learning area planning, among the 14 indicators.

**TABLE 3:** Top five indicators.

Indicator	Overall weight	Overall sequence
Opportunity education	0.131	1
Exercise	0.108	2
Role-play	0.100	3
Team competition	0.095	4
Learning area planning	0.088	5

It is important for teachers to implement crisis awareness education in universities (Sullivan et al., 2011). For this reason, it is necessary to hold crisis awareness courses on campuses regularly and emphasize the differences between each area to plan different course content depending on how little faculty know about crisis management strategies on campus, and how different the dilemmas are in crisis awareness on campuses. Crisis prevention and recognition is the most important element of crisis cognition education. Besides, specific workable crisis response plans should be developed with crisis management manuals for all teachers detailing standard operating procedures for crises, with the definite division of labor and the establishment of a phone tree to quickly complete personnel notification and information transfer during crisis events.

## Data Availability Statement

The original contributions presented in the study are included in the article/supplementary material, further inquiries can be directed to the corresponding author.

## Ethics Statement

The present study was conducted in accordance with the recommendations of the Ethics Committee of the Nanchang University, with written informed consent being obtained from all the participants. All the participants were asked to read and approve the ethical consent form before participating in the present study. The participants were also asked to follow the guidelines in the form in the research. The research protocol was approved by the Ethical Committee of the Nanchang University.

## Author Contributions

LL and XP performed the initial analyses and wrote the manuscript. YH and XL assisted in the data collection and data analysis. All authors revised and approved the submitted version of the manuscript.

## Conflict of Interest

The authors declare that the research was conducted in the absence of any commercial or financial relationships that could be construed as a potential conflict of interest.

## Publisher’s Note

All claims expressed in this article are solely those of the authors and do not necessarily represent those of their affiliated organizations, or those of the publisher, the editors and the reviewers. Any product that may be evaluated in this article, or claim that may be made by its manufacturer, is not guaranteed or endorsed by the publisher.
